# Arterial Response to Shear Stress Critically Depends on Endothelial TRPV4 Expression

**DOI:** 10.1371/journal.pone.0000827

**Published:** 2007-09-05

**Authors:** Veronika Hartmannsgruber, Willm-Thomas Heyken, Michael Kacik, Anuradha Kaistha, Ivica Grgic, Christian Harteneck, Wolfgang Liedtke, Joachim Hoyer, Ralf Köhler

**Affiliations:** 1 Department of Internal Medicine-Nephrology, Philipps-University, Marburg, Germany; 2 Institut für Pharmakologie, Charité Campus Benjamin Franklin, Berlin, Germany; 3 Center for Translational Neuroscience, Duke University, Durham, North Carolina, United States of America; Vrije University Amsterdam, The Netherlands

## Abstract

**Background:**

In blood vessels, the endothelium is a crucial signal transduction interface in control of vascular tone and blood pressure to ensure energy and oxygen supply according to the organs' needs. In response to vasoactive factors and to shear stress elicited by blood flow, the endothelium secretes vasodilating or vasocontracting autacoids, which adjust the contractile state of the smooth muscle. In endothelial sensing of shear stress, the osmo- and mechanosensitive Ca^2+^-permeable TRPV4 channel has been proposed to be candidate mechanosensor. Using TRPV4^−/−^ mice, we now investigated whether the absence of endothelial TRPV4 alters shear-stress-induced arterial vasodilation.

**Methodology/Principal Findings:**

In TRPV4^−/−^ mice, loss of the TRPV4 protein was confirmed by Western blot, immunohistochemistry and by *in situ*-patch–clamp techniques in carotid artery endothelial cells (CAEC). Endothelium-dependent vasodilation was determined by pressure myography in carotid arteries (CA) from TRPV4^−/−^ mice and wild-type littermates (WT). In WT CAEC, TRPV4 currents could be elicited by TRPV4 activators 4α-phorbol-12,13-didecanoate (4αPDD), arachidonic acid (AA), and by hypotonic cell swelling (HTS). In striking contrast, in TRPV4^−/−^ mice, 4αPDD did not produce currents and currents elicited by AA and HTS were significantly reduced. 4αPDD caused a robust and endothelium-dependent vasodilation in WT mice, again conspicuously absent in TRPV4^−/−^ mice. Shear stress-induced vasodilation could readily be evoked in WT, but was completely eliminated in TRPV4^−/−^ mice. In addition, flow/reperfusion-induced vasodilation was significantly reduced in TRPV4^−/−^ vs. WT mice. Vasodilation in response to acetylcholine, vasoconstriction in response to phenylephrine, and passive mechanical compliance did not differ between genotypes, greatly underscoring the specificity of the above *trpv4*-dependent phenotype for physiologically relevant shear stress.

**Conclusions/Significance:**

Genetically encoded loss-of-function of *trpv4* results in a loss of shear stress-induced vasodilation, a response pattern critically dependent on endothelial TRPV4 expression. Thus, Ca^2+^-influx through endothelial TRPV4 channels is a molecular mechanism contributing significantly to endothelial mechanotransduction.

## Introduction

In vascular physiology, Ca^2+^-influx in response to mechanical or agonist stimulation plays a pivotal role in a variety of endothelial functions, in particular in the Ca^2+^-dependent synthesis of endothelium-derived vasodilating factors such as diffusible nitric oxide [1] and prostacyclin [2], as well as the endothelium-derived hyperpolarizing factor (EDHF). EDHF-mediated vasodilation represents a considerable proportion of total vasodilation, which is resistant to inhibitors of nitric oxide- and prostacyclin synthesis, and is caused by endothelium-dependent hyperpolarization of smooth muscle and subsequent closure of voltage-gated Ca^2+^-channels leading to relaxation [3]–[5]. Ca^2+^-permeable cation channels of the transient receptor gene super-family (TRP) [6]–[13] have been proposed to function as Ca^2+^ entry pathway in response to stimulation of G-protein-coupled receptors, as well as to mechanical stimulation by increased flow or shear stress [7], [14]–[16]. Recent studies have provided pharmacological and molecular biological evidence that Ca^2+^-entry mediated by the endothelial TRP vanilloid type 4 channel (TRPV4) is involved in the synthesis of nitric oxide [17] and in EDHF signaling [18]–[20]. TRPV4 channels [16], [21]–[26] can be polymodally activated by osmotic stress [21], [23], [26], shear stress [16], [17], [27], moderate warmth (>27°C) [28], [29], arachidonic acid (AA) and its metabolite 5,6 epoxyeicosatrienoic acid (5,6 EET) [18], [19], possibly by low pH [30], and pharmacologically by the non-PKC-activating phorbol ester, 4α-phorbol-12,13-didecanoate (4αPDD) [21]. *trpv4* gene-targeted mice exhibit a subtle phenotype with deficits in the regulation of systemic tonicity by the central nervous system [24], [31], altered transduction of noxious stimuli [24], [25], [32]–[34], and, amongst other phenotypes, defects in the lung alveolar barrier [35] and in renal tubular K^+^ secretion [36].

We recently presented pharmacological evidence that endothelial TRPV4 may function in signal transduction in response to flow or shear stress [17]. To further analyze TRPV4-mediated mechanisms in vascular endothelial function in a more definitive manner, we now tested shear stress and agonist-induced endothelium-dependent vasodilation in mice lacking the TRPV4 channel [24]. As a result, we demonstrate in carotid arteries from TRPV4^-/−^ mice that (1) these vessels do not relax in response to 4αPDD, (2) shear stress-induced vasodilation is fully eliminated, and (3) flow-induced vasodilation is greatly attenuated, whereas agonist-induced vasodilation and constriction were intact. In summary, our data provide support for an essential role of TRPV4 in the response of arterial endothelia to shear stress.

## Methods

### TRPV4^−/−^ mice, carotid artery preparation, and carotid artery endothelial cells

Genotyping of TRPV4^−/−^ mice [24] was performed by polymerase chain reaction (PCR) of genomic tail DNA. Primers: Forward primer (binds to Exon 11): CGCTTCCTGCTTGTGTACCT; Reverse primer 1 (binds to Exon 13): GGAGTGCCATCTGAGCTCTT. Product size: WT, ∼3.6 kb; TRPV4^−/−^, ∼2.0 kb; Reverse primer 2 (binds to Exon 12): CGATGGTGAGCTTGAAGAGG. Product size: WT, ∼1.7 kb; TRPV4^−/−^, no signal.

To confirm the lack of the TRPV4 protein, we performed immunohistochemistry in carotid arteries (CA) of TRPV4^−/−^ and wild-type mice. Animals were fixed by transcardiac perfusion with 10 % neutral-buffered formalin, post-fixed by overnight immersion in the same fixative, and infused with sucrose. Frozen blocks were sectioned at 12 µm, mounted and immunolabeled using a TRPV4 C-term specific antibody raised in rabbits against a synthetic peptide encompassing positions 855 to 873 of mouse TRPV4 (CDGHQQGYPRKWRTDDAPL), as described previously [24]. Secondary detection was accomplished using a goat anti-rabbit antibody, conjugated to Alexafluor 594 (Invitrogen-Molecular Probes, Carlsbad, CA, USA). Labeled sections of CA were imaged and photographed using an Olympus BX60 upright microscope, RFP filter set, a Roper Coolsnap high-res digital camera, using ISEE image processing software (ISEE, Raleigh, NC, USA).

Western blotting: Experiments were performed using an affinity-purified, polyclonal anti-TRPV4 antibody. The antibody was directed against the synthetic peptide from position 853 to 868 (CDGHQQGYAPKWRTDD) of TRPV4 [37]. In brief, frozen kidneys from each genotype were ground under nitrogen and subsequently homogenized in 50 mM Tris, 1 mM EDTA, 2 mM DTT, 0.2 µM benzamidine, 50 mM leupeptin, 0.5 mM PMSF, 0.1 mg/ml trpysin inhibitor pH 8.0 (homogenization buffer). Debris and nuclear material were removed by centrifugation at 1000×g for 2 min. Membrane proteins were collected by centrifugation at 20,000×g for 45 min, resuspended in homogenization buffer, quantified using the Bio-Rad Protein Assay. 20 µg of membrane proteins were separated by sodium dodecylsulfate–polyacrylamide gel electrophoresis (SDS-PAGE), and electrophoretically transferred to nitrocellulose membranes (Trans-Blot, all from Bio-Rad, Munich, Germany). TRPV4 protein was visualized by an anti-TRPV4 antibody [37], a peroxidase-conjugated anti-rabbit antiserum (GE Healthcare europe, Freiburg, Germany) and ECL advance Western blot detection system (GE Healthcare). Equal loading of proteins was validated by incubation of the Western blot with an anti-tubulin antibody (Labvision, Fremont, CA).

### Electrophysiology

For patch clamp experiments in carotid artery endothelial cells (CAEC) [17], freshly isolated segments of the right and left carotid artery (CA) from female and male TRPV4^−/−^ mice and WT littermates were mounted on a glass capillary with the endothelium facing the bath solution and incubated with 0.05% trypsin and 0.02% ethylenediaminetetraacetic acid (EDTA) in phosphate buffered saline (PBS) without Ca^2+^/Mg^2+^ for up to 10 min, and then washed. For whole-cell patch-clamp experiments, the patch pipette was approached to the luminal surface and a single CAEC was fixed at the tip of the patch pipette by applying a negative pressure (−5 mmHg) to the pipette. After formation of a giga-Ω seal, the cell was carefully detached from the luminal face of the vessel. To achieve electrical access to the cytosol, a negative pressure (∼−50 mmHg) was applied to the pipette leading to rupture of the membrane patch within the tip. Whole-cell membrane currents in CAEC were recorded with an EPC-9 (HEKA) patch-clamp amplifier using voltage ramps (duration: 1000 ms) from −100 to +100 mV as described previously [17]. Initial ohmic leak currents up to 500 pS were subtracted by using the leak correction mode of the EPC9. Cells exhibiting larger leak currents or becoming unstable over time were not further considered. Patch pipettes had tip resistances of 2–4 MΩ in symmetrical KCl solutions. If not otherwise stated, the standard pipette solution was composed of (in mmol/L): 20 CsCl, 100 cesium methane sulfonate, 1 MgCl_2_, 4 Na_2_ATP, 10 EGTA, 0.9 CaCl_2_, 10 HEPES, pH adjusted to 7.2 with CsOH; calculated free [Ca^2+^] was 0.04 µmol/L. The standard NaCl bath solution contained (mmol/L): 137 NaCl, 4.5 Na_2_HPO_4_, 3 KCl, 1.5 KH_2_PO_4_, 0.4 MgCl_2_, 10 glucose, and 1 CaCl_2_ (pH 7.4). In experiments employing hypotonic stress, isotonic and hypotonic bath solutions consisted of (mmol/L): 90 NaCl, 1 CaCl_2_, 1 MgCl_2_,10 glucose, 10 HEPES, pH 7.4. The isotonic solution contained additionally 95 mmol/L mannitol. All experiments were performed at RT. Data analysis was performed as described previously [38].

### Pressure myography

Pressure myography in CA was performed as described previously [17]. Bath and perfusion solutions contained (in mmol/L): 145 NaCl, 1.2 NaH_2_PO_4_, 4.7 KCl, 1.2 MgSO_4_, 2 CaCl_2_, 5 glucose, 2 pyruvate, and 3 MOPS buffer (pH 7.4 at 37°C). CA were mounted on glass capillaries, pressurized to 80 mmHg and were stretched to their *in vivo* length. Initially, CA were continuously perfused at a flow rate of 30 µl/min elicited by a pressure gradient of 1 mmHg between inflow and outflow glass capillaries of equal dimensions. After an equilibration period of 30 min, CAs were pre-constricted with 1 µmol/L phenylephrine (PE) in the bath solution, if not stated otherwise. After development of stable tone, intravascular flow was re-established from almost static conditions (∼30 µl/min) to physiologically relevant levels (600 µl/min) by increasing the pressure gradient between inflow and outflow capillaries to 20 mmHg. The shear stress increased from ∼0.1 to 3 dyne/cm^2^. The mean intraluminal pressure remains constant at 80 mmHg under these conditions. To measure sole shear stress-mediated vasodilation (without increasing flow rate), the viscosity of the perfusion medium was increased from 0.7 to 2.9 mPa*s by adding 5% dextran which enhanced shear stress in CA from ∼3 to 7 dyne/cm^2^. Shear stress was mathematically estimated according to the Hagen-Poiseuille law: τ = 4ηQ/πr^3^; τ = shear stress; η = viscosity; Q = flow, and r = radius. In other sets of experiments, CAs were perfused with 4αPDD (1 µmol/L), or acetylcholine (ACh, 1 nmol/L–10 µmol/L) in the presence and absence of the NO-synthase-inhibitor N^G^-nitro-L-arginine (L-NNA, 300 µmol/L) and the cyclooxygenase inhibitor indomethacin (INDO, 10 µmol/L). To study the dependence of 4αPPD-, shear stress-, as well as flow/reperfusion-induced vasodilation on endothelial intracellular Ca^2+^-signaling, the endothelium was preloaded with the Ca^2+^-chelator 1,2-bis-(*o*-aminophenoxy)ethane-*N*,*N*,*N′*,*N′*-tetraacetic acid tetra-(acetoxy-methyl) ester (BAPTA-AM, 10 µmol/L) in the perfusion solution for 10 min, prior to stimulation. In another subset of experiments, the endothelium was pre-incubated with the PLA_2_ inhibitor 1,1,1-trifluoromethyl-6,9,12,15-heieicosatetraen-2-one (AACOCF_3_, 3 µmol/L) for 10 min. Diameter changes were expressed as a percentage of the maximal dilatation induced by 10 µmol/L sodium nitroprusside (SNP). Maximal constriction was elicited by 60 mmol/L K^+^. Elasticity of CA was tested by increasing the intraluminal pressure in 20 mmHg steps to up to 140 mmHg in the presence of the NO-donor SNP. Chemicals were obtained from Sigma-Aldrich (München, Germany).

### Statistical analysis

Data are given as mean±SEM. The unpaired Students *t*-Test was used to assess differences between groups. *P*-values of <0.05 were considered significant.

## Results and Discussion

### Loss of cationic currents in response to 4αPDD, arachidonic acid and hypotonicity in carotid artery endothelial cells from TRPV4^−/−^ mice

**Figure 1:**
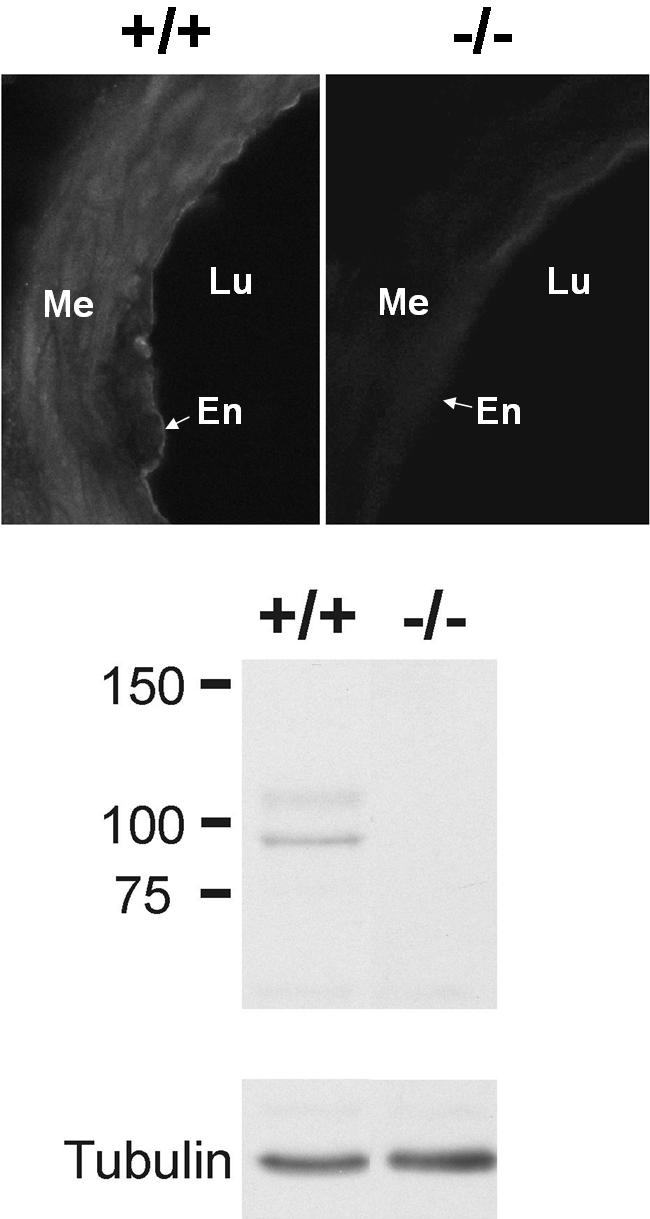
Immunohistochemistry for TRPV4 in CAs (upper panel) and Western blot analysis of TRPV4 protein expression in kidneys (lower panel) of WT and TRPV4^−/−^ mice. Note the absence of specific staining in CA of TRPV4^−/−^ mice. Me = media; Lu = lumen; En = endothelium. In a kidney extract obtained from WT mice, the anti-TRPV4 antibody detected a protein of ∼95 kDa, which is in good agreement with the calculated molecular weight (98 kDa). Furthermore, an additional band of ∼107 kDa, presumably representing the glycosylated protein, is detected by the antibody. In extracts from TRPV4^−/−^ mice, no signals were present. Equal protein loading of the blots was validated by visualization of tubulin.

Expression of the TRPV4 protein was evident in the carotid endothelium and in kidney extracts of WT but not of TRPV4^−/−^ mice as determined by immunohistochemistry and Western blot analysis, respectively (Fig. 1).

**Figure 2:**
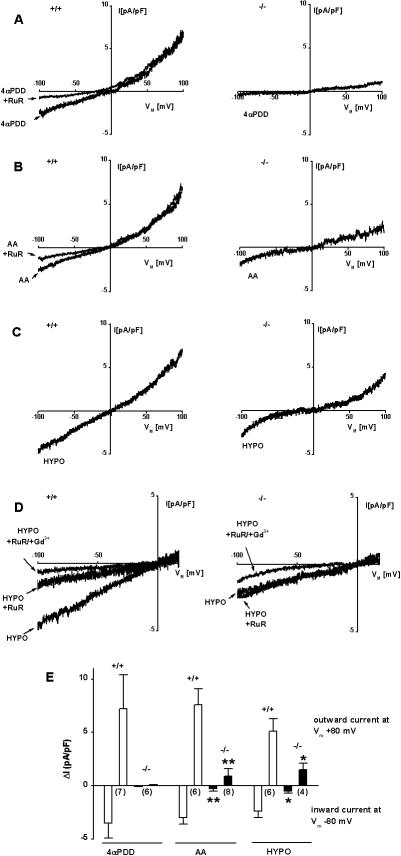
Electrophysiological properties of TRPV4 currents in carotid artery endothelial cells (CAEC) from WT and TRPV4^−/−^ mice. A, left panel, Representative recording of 4αPDD (1 µmol/L)-inducible TRPV4-currents in CAEC of WT. Voltage-dependent inhibition by RuR (1 µmol/L). Right panel, 4αPDD-inducible currents were undetectable in CAEC of TRPV4^−/−^ mice. B, left panel, Representative recording of AA (10 µmol/L)-inducible TRPV4 currents in CAEC of WT and inhibition by RuR (1 µmol/L). Right panel, small AA-inducible cation-currents in CAEC of TRPV4^−/−^ mice. C, left panel, HTS (206 mosmol/L)-inducible TRPV4-currents in CAEC of WT. Right panel, HTS-inducible cation currents of smaller amplitude in CAEC of TRPV4^−/−^ mice. D, left panel, Partial voltage-dependent inhibition of HTS-inducible TRPV4 currents in CAEC of WT and almost complete inhibition by the combination of RuR and Gd^3+^ (10 µmol/L). Right panel, RuR insensitivity and Gd^3+^ sensitivity of HTS-inducible cation currents in CAEC of TRPV4^−/−^ mice. E, Mean 4αPDD-, AA-, and HTS-inducible TRPV4 and other cation currents in CAEC of WT and TRPV4^−/−^ mice. Numbers in brackets indicate the number of cells investigated. Values are given as means±SEM; * P<0.05, ** P<0.01, *t* test.

In whole cell-patch clamp experiments, 4αPDD (1 µmol/L), a synthetic activator of TRPV4, gated moderately outward-rectifying currents in CAEC from WT mice (Fig. 2A left, E). Ruthenium red (RuR, 1 µmol/L, n = 7), a blocker of TRPV channels, reduced inward currents to 10±3% of the initial value, in a voltage-dependent fashion. 4αPDD-inducible currents were absent in CAEC from TRPV4^−/−^ mice (Fig. 2A, right panel, E). Arachidonic acid (AA, 10 µmol/L) produced TRPV4 currents in CAEC of WT littermates, which were reduced by 40±5% by 1 µmol/L RuR (n = 5; Fig. 2B, left panel; E). In CAEC from TRPV4^−/−^ mice, AA generated significantly smaller currents (Fig. 2B; right panel, E). Hypo-osmotic stress (HTS; 206 mosmol/L bath solution, leading to cell swelling and thus stretching of the plasma membrane) activated TRPV4-currents in CAEC of WT littermates (Fig. 2C, left panel). These currents were reduced to 35±5% of the initial value by RuR (n = 6; Fig. 2D, left panel). The remaining RuR-insensitive current was further reduced to 10±4% by 10 µmol/L Gd^3+^ (n = 4), an unspecific inorganic blocker of mechanosensitive channels and a variety of other TRP channels [6]. HTS-induced currents were also observed in CAEC of TRPV4^−/−^ mice (Fig. 2C, right panel), but the amplitude of these currents was significantly reduced compared to those in CAEC from WT animals (Fig. 2E). This smaller HTS-inducible current in CAEC from TRPV4^−/−^ mice was insensitive to RuR, but could be decreased by 70±5% by 10 µmol/L Gd^3+ ^(n = 5; Fig. 2D, right panel), thus indicating that these currents are carried by other channels.

In aggregate, these electrophysiological properties of TRPV4 currents in CAEC of WT mice resemble the characteristics of endothelial TRPV4 from rat carotid artery [17] and mouse aorta [18]. The complete loss of 4αPDD-induced currents in CAEC from TRPV4^−/−^ mice demonstrates the functional expression of TRPV4 in carotid endothelia and also implies that 4αPDD appears to be capable of selectively activating TRPV4 in WT cells. The severely diminished HTS- and AA-induced currents in CAEC from TRPV4^−/−^ mice show that indeed the majority of these currents in WT are carried by endothelial TRPV4. Similar findings were obtained from mouse aortic endothelial cells [18] derived from another TRPV4^−/−^ strain [30], in which TRPV4-activating stimuli such as 4αPDD, AA, AA-metabolites (EETs) and HTS did not produce TRPV4-like currents and TRPV4-associated Ca^2+^-responses [18]. In extension of these findings, we show here that a small residual AA and HTS-inducible cation current, insensitive to RuR but sensitive to Gd^3+^, points to a minor contribution of other yet unidentified AA-sensitive cationic channels and/or HTS-sensitive mechanosensitive channels. In keeping with the RuR-insensitivity of such currents, it appears likely that the deficiency of TRPV4 is not compensated by other closely related members of the TRPV subfamily, such as TRPV1 and TRPV2, which do not seem to be expressed in normal carotid endothelia [17]. In carotid endothelium of either TRPV4^+/+^ or TRPV4^−/−^ mice, TRPV1 does not seem to be considerably expressed and, likewise, the TRPV1 opener capsaicin (1 µmol/L) did not produce endothelium-dependent vasodilation (data not shown). Recently, it has been reported that a member of the two-pore K^+^ family of channels, TREK-1 [39], [40], bears a functional resemblance to TRPV4 and could, thus, be a candidate to substitute for the loss of TRPV4. TREK-1 functions were similar in CAEC of both genotypes (unpublished observation by our group). Thus, these observations further support the idea that loss of TRPV4 is not compensated by other TRPV or TREK channels.

### Vascular compliance in TRPV4^−/−^ mice

**Figure 3:**
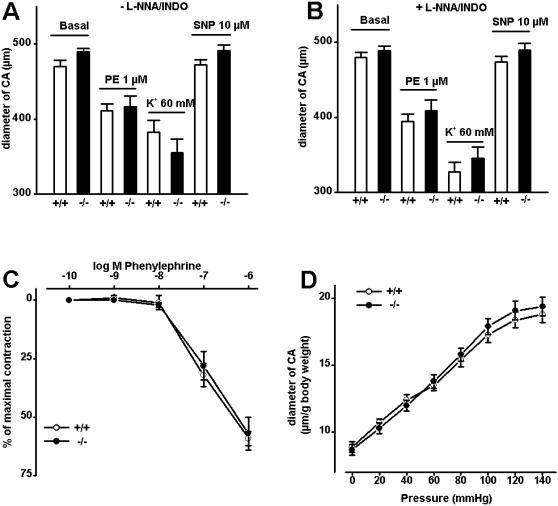
Compliance of carotid arteries (CA) of WT and TRPV4^−/−^ mice. A and B, Diameter of CA pressurized to 80 mmHg (basal) and in the extravascular presence of PE, K^+^, and SNP in the absence and presence of L-NNA (300 µmol/L) and INDO (10 µmol/L); WT CA, -L-NNA/INDO: n = 11 and+L-NNA/INDO: n = 8; TRPV4^−/−^ CA, -L-NNA/INDO: n = 7 and+L-NNA/INDO: n = 8. C, PE-induced contraction of CA from WT (CA, n = 4) and TRPV4^−/−^ mice (CA, n = 4) in the presence of L-NNA and INDO. D, Change in passive diameter (normalized to body weight) of CA from WT (CA, n = 13) and TRPV4^−/−^ mice (CA, n = 10) in response to increasing intravascular pressure and in the presence of SNP. Values are given as means±SEM.

To evaluate the functional role of TRPV4 in the endothelium, we performed pressure myograph experiments in CA from WT and TRPV4^−/−^ mice. In the presence or absence of NO- and cyclooxygenase inhibitors (L-NNA and INDO), the basal (passive) diameter of CA (pressurized to 80 mmHg) from TRPV4^−/−^ mice and WT littermates did not differ (Fig. 3A and B). The constriction in response to either phenylephrine (PE, 1 µmol/L and below) (Fig. 3A, B, and C) or to 60 mmol/L K^+^ (Fig. 3A and B) in TRPV4^−/−^ and WT mice was not statistically different. The vasodilation elicited by sodium nitro prusside (SNP, 10 µmol/L) was similar in both groups (Fig. 3A and B), which thus suggests that TRPV4 deficiency does not compromise endothelium-independent relaxation of smooth muscle. In addition, TRPV4^−/− ^CA and WT CA exhibited a similar passive increase in CA diameter in response to increasing intravascular pressure (range 0–140 mmHg) and in the presence of SNP (to eliminate any myogenic constriction (Bayliss effect) (Fig. 3D). These results indicate that total vascular compliance, elasticity and response to static mechanical pressure from within the lumen of the CA are unaffected by TRPV4 deficiency.

### Loss of 4αPDD-induced vasodilation in TRPV4^−/−^ mice

**Figure 4:**
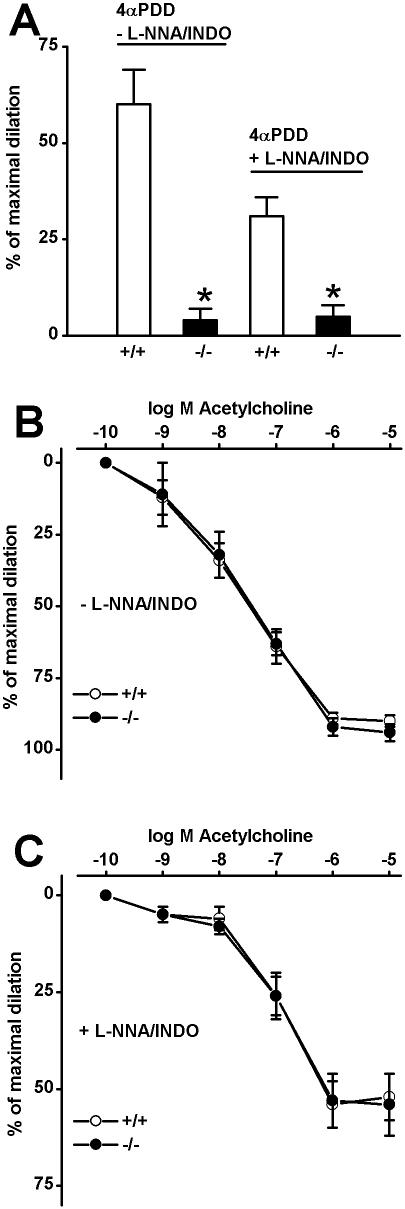
4αPDD and ACh-induced vasodilation in CA of WT and TRPV4^−/−^ mice. A, 4αPDD (1 µmol/L)-induced vasodilation in CA of WT and TRPV4^−/−^ mice in the absence and presence of L-NNA and INDO. WT CA, -L-NNA/INDO: n = 12 and+L-NNA/INDO: n = 9; TRPV4^−/−^ CA, -L-NNA/INDO: n = 6 and+L-NNA/INDO: n = 8. B, ACh-induced vasodilation in CA of WT (n = 7) and TRPV4^−/−^ (n = 6) in the absence of L-NNA and INDO and B, in their presence; WT CA (n = 6); TRPV4^−/−^ CA (n = 6). * P<0.001, *t* test.

Intraluminal application of 4αPDD (1 µmol/L) caused a robust vasodilation of CA (∼60%) in WT mice (Fig. 4A) which was completely absent if 4αPDD was applied together with the TRPV-blocker RuR (1 µmol/L, data not shown). In addition, we tested whether 4αPDD causes EDHF-type vasodilation of WT CA. EDHF-mediated vasodilation is defined as the vasodilation caused by endothelium-dependent hyperpolarization of smooth muscle, which is resistant to inhibitors of NO-synthase and cyclooxygenase. In the presence of both inhibitors, 4αPDD-induced vasodilation was reduced to ∼40% of the value obtained without such inhibitors (Fig. 4A). This results indicates that pharmacological activation of endothelial TRPV4 leads to vasodilation, which is partly caused by an EDHF-type signaling in mouse CA (Fig. 4A), and this is similar to what we observed previously in small arteries from the rat gracilis muscle. However, the 4αPDD-induced response was more pronounced in the murine CA than in the rat CA, in which overall EDHF-signaling is weaker than in murine CA [17], [41], [42].

In murine CA, this 4αPDD-induced EDHF-type vasodilator response was almost abolished by inhibition of endothelial SK_Ca_ and IK_Ca_ channels, the underlying effectors of the EDHF signal in these arteries [41], with UCL 1684 (1 µmol/L) [43] and TRAM-34 (1 µmol/L) [44] (Figure S1). Moreover, this EDHF-type response, as well as the NO/PGI_2_-dependent component, was eliminated after preloading the endothelium with the Ca^2+^-chelator BAPTA-AM to eliminate endothelial Ca^2+^ signaling (Figure S1). This clearly shows that activation of TRPV4 by application of 4αPDD induces vasodilation which critically depends on an increase of intracellular Ca^2+^ within the endothelium.

In striking difference to the WT, a vasodilator-response to 4αPDD could not be observed in TRPV4^−/−^ mice, neither in the absence nor in the presence of NO- and cyclooxygenase inhibitors (Fig. 4A). This strongly suggests that 4αPDD-induced vasodilation in WT mice is mediated by TRPV4, and, in view of the robust amplitude of this vasodilator response, that TRPV4 plays a central role in endothelium-dependent regulation of vascular tone.

### Total acetylcholine-induced vasodilation and EDHF-mediated vasodilation is intact in TRPV4^−/−^ mice

Total acetylcholine-induced vasodilation (in the absence of NO-synthase and cyclooxygenase inhibitors) was unchanged in TRPV4^−/−^ mice vs. WT (Fig. 4B). This was also true for EDHF-mediated vasodilation (Fig. 4C). These data show that TRPV4 is not appreciably involved in acetylcholine-induced and either NO or EDHF-mediated vasodilation. Thus, TRPV4 does not play a role in endothelial agonist-induced and G-protein-coupled receptor-operated Ca^2+^ mobilization and -entry in this conduit artery.

### Loss of shear stress-induced vasodilation in TRPV4^−/−^ mice

The reported mechanosensitivity of TRPV4 channels [14], [16], [25], [27], [34], [45], [46] and the sensitivity of shear stress-induced vasodilation of rat CA and gracilis artery [17] to the TRPV blocker RuR indicate a possible role for TRPV4 in endothelial mechanotransduction.

**Figure 5:**
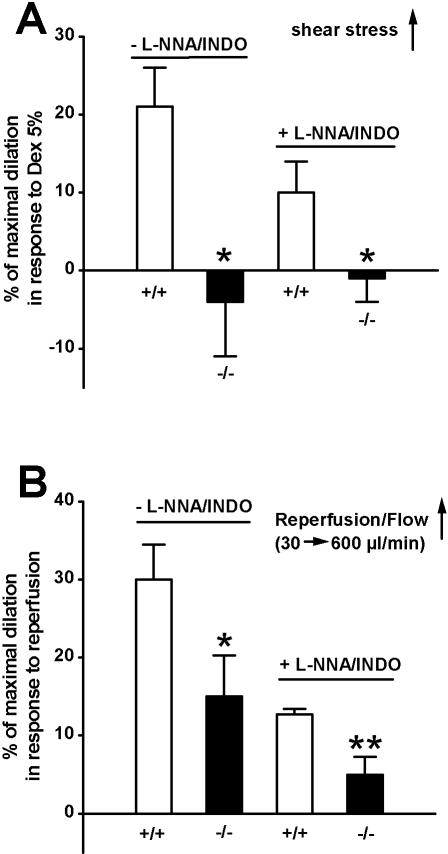
Shear stress- and flow/reperfusion-induced vasodilation in CA of WT and TRPV4^−/−^ mice. A, Shear stress-induced vasodilation in CA of WT and TRPV4^−/−^ mice in the absence and presence of L-NNA and INDO; WT CA, n = 12 and n = 9; TRPV4^−/−^ CA, n = 6 and n = 8, respectively. Shear stress-induced vasodilation was elicited by switching to a perfusion medium containing 5% dextran (5% Dex). B, Flow/reperfusion-induced vasodilation of CA of WT and TRPV4^−/−^ mice in the absence and presence of L-NNA and INDO. WT CA, n = 9 and n = 8; TRPV4^−/−^ CA, n = 7 and n = 7, respectively. * P<0.05, ** P<0.01, *t* test.

To compare shear stress-mediated vasodilation between TRPV4^−/−^ mice and WT littermates, the viscosity of the perfusion medium was increased by addition of 5% dextran, resulting in a physiologically relevant increase in shear stress from ∼3 to 7 dyn/cm^2^. In WT mice, the increase in viscosity elicited a vasodilation of ∼20% in the absence of NO- and cyclooxygenase inhibitors (Fig. 5A). Inhibition of cyclooxygenases did not reduce this response suggesting that prostacyclin production does not contribute to this vasodilator response (data not shown). In the presence of both inhibitors, the increase in shear stress resulted in a diminished vasodilation of ∼10%, exclusively mediated by the EDHF system (Fig. 5A). This EDHF-type vasodilator response was almost abolished by inhibition of endothelial SK_Ca_ and IK_Ca_ channels (Figure S2). Buffering of endothelial Ca^2+^ greatly attenuated both the EDHF response alone, as well as the composite NO/EDHF response (Figure S2), a similar effect to that on 4αPDD responses. Like shear stress-induced vasodilation in small and large arteries of the rat [17], murine CA vasodilation was blocked by the TRPV inhibitor RuR or by pre-incubation with 3 µmol/L AACOCF_3_ (Figure S2) to prevent the release of AA and production of AA metabolites, the putative endogenous activators of TRPV4 [18], [19]. This dependency on PLA_2_ activity - also shown by others recently [20] - possibly implies that products of PLA_2_ can enhance the mechanotransductory function of TRPV4. Alternatively, TRPV4 channels may not function directly as the mechanosensor, rather as critical components of down-stream signaling (reviewed in Liedtke and Kim 2005).

Strikingly, in TRPV4^−/−^ mice, the dextran-mediated increase in shear stress did not cause any NO or EDHF-mediated vasodilation, but rather resulted in a subtle, yet appreciable vasoconstriction (Fig. 5A). This complete loss of shear stress-induced vasodilation in TRPV4^-/− ^mice demonstrates that Ca^2+^-entry through TRPV4 is a crucial step in endothelial mechanotransduction in response to shear stress, not compensated by any other gene in the absence of TRPV4.

In the present study, we go on to demonstrate that CA of TRPV4^−/−^ mice exhibit an altered vasodilation in response to re-establishing flow through the vessel (reperfusion) from almost static conditions (30 µl/ml) to physiologically relevant levels (600 µl/ml), when compared to WT littermates. In general, flow-evoked vasodilation is a complex Ca^2+^-dependent (as well as possibly Ca^2+^-independent), NO- and EDHF-mediated and strictly endothelium-dependent process [47]–[49], which in these respects is remarkably similar to shear-stress induced vasodilation. In WT mice, the onset of flow, as happens in reperfusion, caused vasodilation of ∼30% in the absence of NO- and cyclooxygenase inhibitors (Fig. 5B). Similar to shear stress-induced vasodilator responses, this flow/reperfusion-evoked vasodilation was on average reduced to ∼10% in the presence NO and cyclooxygenase inhibitors (Fig. 5B), thus indicating a partial contribution of the EDHF system. Consequently, dual inhibition of endothelial SK_Ca_ and IK_Ca_ channels eliminated this EDHF response (Figure S3). Inhibition of cyclooxygenases alone did not diminish this response suggesting that prostacyclin production does not add further to this flow/reperfusion-induced vasodilation in the murine carotid artery (data not shown). Again, like for shear stress and 4αPDD responses, buffering of endothelial Ca^2+^ virtually eliminated the EDHF response alone and greatly reduced the composite NO/EDHF response (Figure S3). Moreover, inhibition of PLA_2_ by AACOCF_3_ abolished flow/reperfusion-induced and NO/EDHF-mediated vasodilation (Figure S3), thus indicating that the release of AA and generation of AA metabolites, possibly functioning as (direct or indirect) endogenous modulators of TRPV4, are also important in this type of vasodilation.

In CA of TRPV4^−/−^ mice vs. WT, flow/reperfusion resulted in a significantly reduced vasodilation of ∼15% and ∼5% in the absence and presence of NO and cyclooxygenase inhibitors, respectively (Fig. 5B). Therefore, these data suggest that TRPV4 also participates critically in flow/reperfusion-induced vasodilation, which, however, in contrast to shear stress-induced vasodilation, is not completely dependent on TRPV4 and may involve other Ca^2+^-dependent or possibly Ca^2+^-independent mechanisms [49].

In conclusion, we used genetically engineered TRPV4^−/−^ mice to demonstrate the absolute dependence of vasodilation in response to physiologically relevant shear stress on endothelial TRPV4 and a significant contribution of TRPV4 to flow/reperfusion-induced vasodilation. These novel findings are based on the following facts: (1) The TRPV4-activator 4αPDD dilated WT CAs, a response-pattern completely absent in TRPV4^−/−^. (2) Shear stress-induced vasodilation was also completely absent in TRPV4^−/−^ mice. (3) Flow/reperfusion-induced vasodilation was greatly diminished in TRPV4^−/−^ mice. In contrast, smooth-muscle dependent SNP-mediated vasodilation and acetylcholine-induced vasodilation did not differ between TRPV4^−/−^ and WT mice, reiterating the specificity of our findings (1–3). Based on our observations, a novel concept emanates, namely that mechanical activation of TRPV4 in arteries by physiologically relevant shear stress and flow/reperfusion is a critical component of endothelial mechanotransduction.

On a final note, this novel role of TRPV4 not only advances our basic understanding of vascular physiology, it also renders TRPV4 an appealing target [50], [51] for therapeutic manipulation of arterial diameter, e.g. in cardiovascular disease states like hypertension [52], ischemia-reperfusion-induced vascular injury, and, last but not least, in atherosclerosis, where chronic mechanical shear stress and inappropriate endothelial Ca^2+^ influx have been suggested to be pathogenic for regional lesion development and progression (e.g. at the carotid bifurcation). For atherosclerosis, it is tempting to speculate that the inflammatory component of the process sensitizes endothelial TRPV4 via cytokine-mechanisms [25]. At least as attractive, and not mutually exclusive is the hypothesis that arterial injury, like in early atherosclerosis, will up-regulate endothelial expression of proteinase-activated-receptor 2 [53], which will be activated proteolytically within the atherosclerotic lesion and via systemic proteases to specifically sensitize endothelial TRPV4 channels to respond to mechanical stress [34].

## Supporting Information

Figure S1 Pharmacological properties of 4αPDD-induced vasodilation in murine CA. From left to right: Inhibition of EDHF-type responses (in the presence of L-NNA and INDO) by the combination of IK^Ca^/SK^Ca^-blockers (TRAM-34 (1 µmol/L) and UCL 1684 (1 µmol/L); n = 4) and after preloading the endothelium with BAPTA-AM (10 µmol/L; n = 6) for 10 min to buffer intracellular Ca^2+^. Inhibition of composite NO/EDHF-type responses (in the absence of L-NNA and INDO) by BAPTA-AM (n = 4). For controls (ctrl, white bars), values±SEM are given in figure 4. * P<0.05, t test.
(0.12 MB TIF)

Figure S2 Pharmacological properties of shear stress-induced vasodilation in murine CA. From left to right: Inhibition of shear stress (Dex 5%)-induced EDHF-type responses by IK^Ca^/SK^Ca^ blockers (n = 11), after buffering of endothelial intracellular Ca^2+^ with BAPTA-AM (n = 7). Inhibition of composite NO/EDHF-type responses by BAPTA-AM (n = 8), the TRPV4 blocker RuR (1 µmol/L; n = 4), and by the PLA^2^ inhibitor AACOCF^3^ (3 µmol/L; n = 4). For controls (ctrl, white bars), values±SEM are given in figure 5 A. * P<0.05, t test.
(0.10 MB TIF)

Figure S3 Pharmacological properties of flow/reperfusion-induced vasodilation in murine CA. From left to right: Inhibition of EDHF-type responses by IK^Ca^/SK^Ca^ blockers (n = 5), after buffering of endothelial intracellular Ca^2+^ with BAPTA-AM (n = 3). Inhibition of composite NO/EDHF-type responses by BAPTA-AM (n = 7), and by the PLA^2^ inhibitor AACOCF^3^ (n = 8). For controls (ctrl, white bars), values±SEM are given in figure 5 B. * P<0.05, t test.
(0.12 MB TIF)
